# Effectiveness of Positive Thinking Training Program on Nurses' Quality of Work Life through Smartphone Applications

**DOI:** 10.1155/2017/4965816

**Published:** 2017-05-14

**Authors:** Mohadeseh Motamed-Jahromi, Zhila Fereidouni, Azizallah Dehghan

**Affiliations:** ^1^Department of Medical and Surgical Nursing, School of Nursing, Fasa University of Medical Sciences, Fasa, Iran; ^2^Noncommunicable Diseases Research Center, Fasa University of Medical Sciences, Fasa, Iran

## Abstract

**Aim:**

Job stress is a part of nurses' professional life that causes the decrease of the nurses' job satisfaction and quality of work life. This study aimed to determine the effect of positive thinking via social media applications on the nurses' quality of work life.

**Methods:**

This was a pretest-posttest quasi-experimental study design with a control group. The samples were selected among the nurses in two hospitals in Fasa University of Medical Sciences and divided randomly into two interventional (*n* = 50) and control (*n* = 50) groups. Positive thinking training through telegrams was sent to the intervention group during a period of 3 months. Data were collected by using Brooks and Anderson's questionnaire of work life quality and analyzed by SPSS 18.

**Results:**

The mean total scores of pretest and posttest in the intervention group improved noticeably and there were significant differences between mean scores of quality of work life in pretest and posttest scores in interventional groups (*p* < 0.001) and in dimensions of work life quality, home life (*p* < 0.001), work design (*p* < 0.001), work context (*p* < 0.001), and work world (*p* = 0.003).

**Conclusion:**

This study concluded that positive thinking training via social media application enhanced nurses' quality of work life. This study is necessary to carry out on a larger sample size for generalizing findings better.

## 1. Introduction

Job stress is an inseparable part of nurses' professional life that occurs when nurses do not have the decision-making ability and skill levels to encounter the demands of the job [1] and may lead to low job satisfaction, somatic and mental disorders, and poor clinical practice of nurses [2]. Nurses' job stress is due to heavy workload, inadequate staff, large number of patients, lack of administrative support, changing healthcare environment, and emotional challenges of working with ill and dying patients [3]. Such problems affect the life quality of nurses as well as their job satisfaction, which ultimately lead them to maintain or abandon their jobs [4].

Quality of working life reflects the attitude and feeling of workers towards their jobs [5]. It is defined as a concept which suggests that an employee's professional needs are fulfilled in the workplace where the organizational goals are achieved [6]. This concept illuminates the impact of the workplace on job satisfaction, content/discontent with home life, overall satisfaction with life, personal happiness, and mental wellbeing [7]. Factors affecting the quality of professional life, based on Walton theory, are fair and adequate income, safe and healthy working environment, providing opportunities for growth and continuous security, rule of law within the organization, social dependence of work life, whole space of work life, social integration and unity of the organization, and the development of human capabilities [8]. Quality improvement of work life can reduce the desire to leave the nursing profession [9].

One of the techniques that can be effective in improving the function and reducing job stress of nurses is positive thinking and optimism. This technique has an impact on the behavior of people to cope with stressful experiences and overcome them [10]. A positive person evaluates stressful situations with optimism and has a good estimation of his/her ability to serve the ripples and difficulties of life [11]. Growing optimism in life increases the productivity of one's opinions and shifts his/her positive thoughts, intellectual properties, and behaviors towards a positive direction. Positive thinking encourages people to be persistent, effectively pursue their goals, and take steps to optimize the quality of their life [12]. The positive psychological interventions include therapies or activities intended to foster positive emotions, positive behaviors, positive perception, and knowledge, enhance wellbeing, and improve the symptoms of depression [13]. A study has shown that positive thinking and optimism have a positive relationship with nurses' professional commitment, which increases the patients' satisfaction and improves job performance [14]. Researchers in a study concluded that positive thinking and optimism interventions reduce job burnout and thus play a fundamental role in decreasing emotional exhaustion and increasing individual efficiency. More optimist nurses are less likely to show emotional anger and fatigue [15]. Another research suggests that positive thinking training enhances the ability and competence of nurses and will lead to positive recognition and reinforcement of positive behaviors [13]. One study emphasized that teaching positive thinking techniques reduces job stress and improves the quality of life [16]. To teach various techniques to reduce stress and increase compatibility, face-to-face classroom teaching is used. Despite all its benefits, it seems that it is difficult for nurses to take part in such classes due to heavy workload and working shifts of nurses. Therefore, using a more accessible tool for education has been of interest to researchers of this study. Currently, using smartphone applications as a means for learning has caught the attention of educators. The advantages include popularity, portability, accessibility, reliability, and saving time and money [17]. Given that use of smartphone social applications such as WhatsApp and Telegram has made communication quick, doing collective teaching with such applications is rather easy. According to description, it seems that the selection of an appropriate and updated instructional method can lead to increased training efficiency. So, the researchers in this study decided to investigate the impact of teaching positive thinking via using social networking applications on the lifework quality of nurses. Based on researchers' searching in different databases such as PubMed, Scopus, Web of Science, and Google Scholar, there was no definite evidence on the effect of positive thinking training program via using smartphone social networking applications on dimensions of nurses' quality of work life. Thus, this study examines this effectiveness. The present study tests this hypothesis: positive thinking training program via social media applications has a constructive effect on dimensions of nurses' quality of work life (home life, work design, working context, and work world) when demographic variables have been taken into account.

## 2. Materials and Methods

### 2.1. Research Design

This study uses an experimental pretest-posttest method on a control group with specific focus on the impact of teaching positive thinking skills on the quality of work life of nurses working in educational hospitals of Fasa University of Medical Sciences in the south of Iran in 2016. The inclusion criteria are as follows: nurses with at least a bachelor's degree and a working background of at least 6 months of nursing care with no history of mental and physical diseases before working as a nurse. History of certain diseases (bipolar disorders, clinical depression, and panic and anxiety disorders) before getting employed and lack of will to participate in the study were chosen as the exclusion criteria.

### 2.2. Participants' Characteristics

The sample size for participants in the intervention and control groups was 100 based on confidence level of 95%, power of test 90%, mean difference 30 in the study's groups, and standard deviation 45 in a previous study [18]. Both the intervention and the control groups had 50 participants. The participants in the intervention group were randomly chosen from nurses equipped with smartphone and social networking applications and also the control group was randomly selected by matching age (3 categories) and sex. To avoid association with the other group, the control group participants were selected from hospital 2.

### 2.3. Intervention

The nurses in the intervention group were given similar contents and messages of positive thinking skills, motivation, and happy songs (Box 1). These messages were sent by different types of media including video, audio, and picture messages via a social networking application (Telegram). Positive thinking quotes and picture messages were sent to nurses more than video and audio. On average, 12 picture messages, 3 audios, and 1 video were sent daily. The participants could send a positive message if they wanted them in the Telegram group. The intervention group compared to the control group received paper-based information only. Nurses in the control group could use positive thinking books placed on the bookshelves in clinical units of hospital 2 in their free time. This training program lasted 3 months and posttest data was gathered no more than 1 week after completion of the training period in the two groups.

### 2.4. Ethical Consideration

This study was approved under project number 93144 in Fasa University of Medical Sciences, Vice Chancellor of Research. The ethics code is IR.FUMS.REC.1395.87. After observing the ethical issues of the research, obtaining the consent of the head nurses, and getting voluntary consent of nurses, the questionnaires were given to the nurses to fill personally. It was attempted to explain the research goals to the nurses. The participants were assured that their personal information remained secret and the questionnaires would be filled anonymously and they could choose to withdraw from the study at any time. The time for the completion of the questionnaires did not interfere with the working hours of nurses, and the nurses filled them in in their free time.

### 2.5. Measures and Covariates

According to the study goals, two questionnaires were used to collect data. The first questionnaire asked for demographic information (age, sex, marital status, education, working hours, overtime working hours, work experience, turnover, and use of sedative drugs and drinks). The second part was Brooks and Anderson's Quality of Work Life questionnaire that included four domains of quality of working life with 42 questions. Out of these 42 questions, 7 questions address home life, 10 are concerned with work design, 20 questions were related to the working context, and 5 questions were concerned with work world [19]. The responses were coded using five-point Likert scale from 1 (completely disagree) to 5 (completely agree). Only 20 alternatives in this questionnaire were reversely coded. The least score was 42 and the highest was 210. High scores showed a better quality of work life. The translation process of the questionnaire into Persian included two forward translations and two back translations each by two bilingual translators. The content validity of the scale was confirmed by a panel of experts in nursing. The relevance, clarity, and simplicity of each item were assessed using the CVI score. Face validity was carried out with 30 voluntary participants. Cronbach's alpha showed that the reliability of the questionnaire was 0.84. Cronbach's *α* coefficients for the different domains were as follows: home life (7 items), 0.85; work design (10 items), 0.90; working context (20 items), 0.78; work world (5 items), 0.84. For data analysis, SPSS version 18 was used. The mean and standard deviation were used to describe quantitative data, and frequencies were used to describe qualitative data. To compare within-group mean scores of quality of work life before and after the intervention, Wilcoxon nonparametric test was used. To compare the scores of pretests and posttests of the control group and intervention group, Mann–Whitney *U* test was used. Analysis of covariance was carried out to statistically control the effect of pretest on posttest scores.

## 3. Results

The data analysis showed that the age range of the nurses in this study was between 22 and 55 years, and their mean age was 30 years.  Table 1 shows the nurses' demographic characteristics together with the comparison of mean and standard deviation in test and retest of the control and intervention group based on demographic variables. As can be seen, the difference between the mean scores of nurses' work life quality working in different wards (*p* = 0.08) was significant. Furthermore, using or not using sedative drugs (*p* = 0.011) was also a significant factor in work life quality scores among the nurses. The findings in Table 2 show that the difference between the total mean scores of pretest and posttest in the intervention group is more than in the control group. Also, there was a significant difference between the total mean scores of pretest and posttest in the intervention group (*p* < 0.001). Furthermore, there was a significant difference between pretest and posttest scores in the intervention group in dimensions of work life (*p* < 0.001), work design (*p* < 0.001), work context (*p* < 0.001), and work world (*p* = 0.003). Analysis of covariance (ANCOVA) showed a significant difference among the means of the pretest and posttest scores in intervention and control groups. Table 2 illustrates the results of ANCOVA.

## 4. Discussion

The major finding of this study showed that the difference between the total mean scores of pretest and posttest in the intervention group was noticeable more than in the control group. The results of the study confirmed that interventions using social media tools were associated with improved knowledge, attitudes, and skills [20]. Based on the results, there was a significant difference between the scores of pretest and posttest in the intervention group. This revealed that the mean of work life quality of the nurses increased after receiving the training. Therefore, one can conclude that teaching positive thinking has a positive impact on work life of nurses. The results of a study by Amponsah-Tawiah and Mensah are compatible with the findings of this study and reveal that factors such as increased self-efficacy, optimism, and flexibility can improve the quality of work life [21]. Researchers in one study criticized the full impact of positive thinking teaching programs and argued that cognitive-behavioral methods and treatments based on responsibility have a higher impact on the increase of power and life quality of individuals [22]. Another finding of this study revealed the significant difference between pretest and posttest scores with respect to home life. This finding shows that teaching positive thinking has a positive impact on home life of nurses. This finding is in line with a research done by Zhang et al., who proposed that teaching positive thinking and hopeful thoughts is reflected in the recipients' behavior, feeling, and thinking, which raises satisfaction of home life [23]. No research has so far rejected this finding.

This study also showed that there was a significant difference between the scores of pretest and posttest in the intervention group with respect to work life. This finding displays that teaching positive thinking has a positive impact on nurses' work life. They learn to remain hopeful about the future, and this will lead to their job satisfaction [24]. In order to explain this finding, first we need to explain that this dimension, as a matter of fact, reveals the nature of being a nurse and its relevant problems including job satisfaction level, heavy workload, lack of professional independence, lack of time and colleagues, job abandonment, and giving service to patients [19, 25]. They learn to remain hopeful about the future, and this will lead to their job satisfaction.

Another finding showed a significant difference between the scores of pretest and posttest in the intervention group with respect to work context, which showed an improvement as a result of positive thinking teaching. The findings of a study by Kluemper et al. support this finding and state that positive thinking and optimism increase organization commitment and enhance organizational behavior [26]. Another study also emphasizes that optimism affects the job performance of employers and increases their sense of wellbeing. Furthermore, it improves their attitude towards work, creativity, and problem-solving competence [27, 28]. Another study reveals that when someone experiences optimism, he/she becomes motivated in doing difficult tasks with higher spirit and satisfaction. Such a person has a greater desire to fulfill his/her goals and is more resistant against problems. When faced with failures, he/she analyzes them and uses them as an opportunity [29]. Work context explains the impact of working environment on nurses and patients and also includes different situations and the ways to deal with colleagues. Work context also includes opportunities for progress and organizational atmosphere [19].

One aspect of work life quality is work world affected by positive thinking training in the intervention group. This aspect illuminates wide impacts of the society on nursing performance, including attitude to society to nursing profession, job security, market, income range, and trust in nursing as a profession [19]. As far as the authors of this article searched, there has been no other research to confirm this finding. It should be stated that positive thinking also raises self-esteem and quality of patient care with a sense of purpose and belief in a bright future, and it is also helpful to achieve organizational goals. Finally, it leads to job security for positive thinking nurses. On the other hand, with a better understanding of their job, job progress, and perfect patient care, nurses will be able to play a fundamental role in shaping and improving the attitude of society towards nursing.

The difference between the means of work life qualities related to different wards was also significant. It was determined that those nurses who worked in special wards were more affected by positive thinking training. The results of Augusto-Landa et al. are in accordance with our research. They found that occupational stress of nurses working in the ICU was overcome after providing training on their psychological empowerment skills [10]; that is, nurses in special wards experience a high level of stress [11]. Every day, they face death, suffering, and pain of their patients and mourning of those around them, and thus they are under a lot of psychological pressure, and teaching positive thinking can become handy in such stressful job with lots of tension. Positive thinking gives nurses' life meaning and freshness and improves their work life quality.

Another finding of this study is that there was a significant difference between the scores of nurses who took sedative drugs and those who did not. In this study, it was also found that those who used tranquilizers were more influenced by positive thinking. According to our search, no similar findings were found. To explain more, nurses who used medical or herbal tranquilizers were aware of their psychological distress, and thus they tried to solve the problem. They are in need of treatment and education more than others. Therefore, under the influence of positive psychological training, their job distress was controlled, and their quality of work life was improved.

## 5. Conclusion

The goal of this study was to add to the importance and the possibilities that social media applications have in positive thinking training program. This research concluded that the use of social media training allows nurses to interact with positive thinking contents and causes an improvement in the nurses' quality of work life. Additionally, potential training happens regardless of place. So, using social media training can be a worthwhile resource and an appropriate alternative or support to traditional training, which may bring about a great return on a small investment of time and money. It is expected that the results of the study can provide a simple and low-cost solution for authorities and nurse managers. It is important to note that the nursing shortage and heavy workload of nurses cause dissatisfaction, disappointment, and burnout in Iranian nurses. Continuous positive thinking training on social media has a true impact on reducing nurses' job stress, depression, and burnout. So, the nurse managers can provide a proper ground for using social media training to improve the conditions of nurses. Using social media in online positive training opens up opportunities for nurses, especially when it comes to peer-based feedback and support. Online support, advice, and feedback all help improve their motivation. In short, providing positive thinking training via social media applications by nurse managers can even cultivate a sense of community, which makes nurses feel like they are part of something bigger, and support with their managers. The end result of having such training is undoubtedly improvement of job satisfaction and burnout decrease.

The limitations of this research included the limited research population and using only one method (questionnaire) to measure the variables. Therefore, to have more generalizable findings, one suggestion is to carry out similar studies in a wider geographical area with larger sample sizes and with a qualitative method. Another limitation of the study was collecting posttest data 1 week after completion of the training program that did not show a long-term effect. We recommend collecting posttest data after several weeks in similar studies.

## Figures and Tables

**Box 1:**
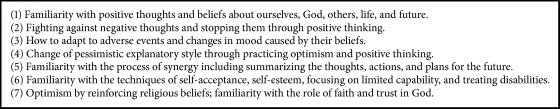
The content of the education package.

**Table 1:** Comparison of quality of work life's mean based on demographic variables in the control and intervention group.

Background characteristics	Control group				Intervention group			
	*n*	%	Mean	*p* value	*n*	%	Mean	*p* value
Age (year)				0.52				0.2
<30	33	66	114.40 ± 33.55		27	54	123.35 ± 24.49	
30–40	15	30	114.84 ± 21.11		17	34	125.09 ± 23.08	
>40	2	4	127 ± 27.32		6	12	139.50 ± 24.27	
Gender				0.16				0.08
Female	40	80	106.77 ± 16.69		42	84	127.13 ± 24.95	
Male	10	20	117.47 ± 31.46		8	16	116.38 ± 18.34	
Marriage				0.75				0.31
Single	33	66	87.19 ± 10.87		34	68	127.58 ± 22.72	
Married	17	34	86.55 ± 9.37		16	32	122.71 ± 25.59	
Education				0.56				0.09
B.S.	48	96	115.11 ± 29.84		46	92	124.18 ± 23.74	
M.S.	2	4	112.33 ± 26.29		4	8	141.16 ± 27.69	
Nursing work experience (year)				0.73				0.21
6 m–10 y	13	26	113.30 ± 46.33		14	28	117.84 ± 27.23	
10–20 y	27	54	117.96 ± 18.74		23	46	128.32 ± 20	
20–30 y	10	20	113.95 ± 25.77		13	26	127.87 ± 27.88	
Ward				0.7				0.008
Critical wards	7	14	121.68 ± 21.26		18	36	136.64 ± 21.26	
General wards	17	34	114.22 ± 36		32	64	124.28 ± 24.45	
Mental wards	26	52	112.15 ± 22.09		0			
Use of sedative drugs and drinks				0.014				0.011
Yes	4	8	131.47 ± 49.30		13	26	138.64 ± 25.76	
No	46	92	112.28 ± 22.72		37	74	122.44 ± 23.04	

**Table 2:** Means and standard deviation of quality of work life's dimensions in pretest and posttest of control and intervention group.

Quality of work life dimensions	Pretest	Posttest	*p* value^*∗*^	ANCOVA		
				df	*f*	*p* value^*∗∗*^
Home life						
Intervention	5.11 ± 19.70	4.16 ± 22.84	<0.001	2	80.80	<0.001
Control	4.63 ± 18.74	4.31 ± 19.14	0.151	1	118.21	<0.001
*p* value^*∗∗∗*^	0.23	<0.001				
Work design						
Intervention	26.10 ± 6.59	30.68 ± 5.39	<0.001	2	77.51	<0.001
Control	26.22 ± 5.28	26.12 ± 5.48	0.682	1	116.70	<0.001
*p* value^*∗∗∗*^	0.83	<0.001				
Work context						
Intervention	59.38 ± 31.86	62.16 ± 12.03	<0.001	2	17.60	<0.001
Control	50.78 ± 13.82	51.18 ± 13.52	0.026	1	14.32	<0.001
*p* value^*∗∗∗*^	0.20	<0.001				
Work world						
Intervention	15.50 ± 3.96	16.60 ± 3.42	0.003	2	125.88	<0.001
Control	14.68 ± 3.88	15.10 ± 3.46	0.05	1	235.65	<0.001
*p* value^*∗∗∗*^	0.59	0.08				
Total score						
Intervention	120.68 ± 34.40	132.28 ± 19.89	<0.001	2	118.31	<0.001
Control	110.42 ± 23.01	111.54 ± 22.43	0.018	1	78.59	<0.001

^*∗*^ *p* value = Wilcoxon.

^*∗∗*^ *p* value = ANCOVA.

^*∗∗∗*^ *p* value = Mann–Whitney.

Significant at the level of *p* < 0.05.

## References

[1] Schaufeli W. B., Bakker A. B., van Rhenen W. (2009). How changes in job demands and resources predict burnout, work engagement, and sickness absenteeism. *Journal of Organizational Behavior*.

[2] Pulido-Martos M., Augusto-Landa J. M., Lopez-Zafra E. (2012). Sources of stress in nursing students: a systematic review of quantitative studies. *International Nursing Review*.

[3] Patterson S. L. (2016). The effect of emotional freedom technique on stress and anxiety in nursing students: a pilot study. *Nurse Education Today*.

[4] Rosser V. J., Javinar J. M. (2003). Midlevel Student Affairs Leaders’ Intentions to Leave: Examining the Quality of Their Professional and Institutional Work Life. *Journal of College Student Development*.

[5] Holden R. J., Scanlon M. C., Patel N. R. (2011). A human factors framework and study of the effect of nursing workload on patient safety and employee quality of working life. *BMJ Quality and Safety*.

[6] Swamy D. R., Nanjundeswaraswamy T., Rashmi S. (2015). Quality of work life: scale development and validation. *International Journal of Caring Sciences*.

[7] Sirgy M. J., Efraty D., Siegel P., Lee D-J. (2001). A new measure of quality of work life (QWL) based on need satisfaction and spillover theories. *Social Indicators Research*.

[8] Nayeri N. D., Salehi T., Noghabi A. A. A. (2011). Quality of work life (QWL) and productivity among Iranian nurses. *Contemporary Nurse*.

[9] Moslem H., Hamid A., Ghanbar R., Alireza S., Hossein N. (2012). Assessing the relationship between nurses' quality of work life and their intention to leave the nursing profession. *Nursing Management*.

[10] Augusto-Landa J. M., Pulido-Martos M., Lopez-Zafra E. (2011). Does perceived emotional intelligence and optimism/pessimism predict psychological well-being?. *Journal of Happiness Studies*.

[11] Nes L. S., Segerstrom S. C., Sephton S. E. (2005). Engagement and arousal: optimism's effects during a brief stressor. *Personality and Social Psychology Bulletin*.

[12] Froman L. (2010). Positive psychology in the workplace. *Journal of Adult Development*.

[13] Sin N. L., Lyubomirsky S. (2009). Enhancing well-being and alleviating depressive symptoms with positive psychology interventions: a practice-friendly meta-analysis. *Journal of Clinical Psychology*.

[14] Luthans K. W., Lebsack S. A., Lebsack R. R. (2008). Positivity in healthcare: Relation of optimism to performance. *Journal of Health, Organisation and Management*.

[15] Garrosa E., Moreno-Jiménez B., Rodríguez-Muñoz A., Rodríguez-Carvajal R. (2011). Role stress and personal resources in nursing: A cross-sectional study of burnout and engagement. *International Journal of Nursing Studies*.

[16] Kooshalshah S. F. R., Hidarnia A., Tavousi M., Shahmohamadi S., Aghdamizade S. (2015). Effect of positive thinking intervention on the nurses’ job stress. *Acta Medica Mediterranea*.

[17] Moreno-Villalon A., Guerra-Martin A., Boemo E. MiniFPGA: An educational app for teaching partitioning, placemnent and routing on Andriod devices.

[18] Navidian A., Saber S., Rezvani AM., Kianian T. (2014). Correlation of quality of work life and job satisfaction in nurses of. *Journal of Health Promotion Management*.

[19] Brooks B. A., Anderson M. A. (2005). Defining quality of nursing work life. *Nursing Economics*.

[20] Cheston C. C., Flickinger T. E., Chisolm M. S. (2013). Social media use in medical education: a systematic review. *Academic Medicine*.

[21] Amponsah-Tawiah K., Mensah J. (2014). Work stress and quality of work Life: The mediating role of psychological capital research. *Journal in Organizational Psychology and Educational Studies (RJOPES)*.

[22] Collins S. (2008). Statutory social workers: Stress, job satisfaction, coping, social support and individual differences. *British Journal of Social Work*.

[23] Zhang A., Cui L., Iyer A., Jetten J., Hao Z. (2014). When reality bites: Hopeful thinking mediates the discrimination-life satisfaction relationship. *Analyses of Social Issues and Public Policy*.

[24] Almalki M. J., FitzGerald G., Clark M. (2012). The relationship between quality of work life and turnover intention of primary health care nurses in Saudi Arabia.. *BMC health services research*.

[25] Reeves R., West E., Barron D. (2005). The impact of barriers to providing high-quality care on nurses' intentions to leave London hospitals. *Journal of Health Services Research and Policy*.

[26] Kluemper D. H., Little L. M., Degroot T. (2009). State or trait: Effects of state optimism on job-related outcomes. *Journal of Organizational Behavior*.

[27] Avey J. B., Luthans F., Smith R. M., Palmer N. F. (2010). Impact of positive psychological capital on employee well-being over time. *Journal of Occupational Health Psychology*.

[28] Culbertson S. S., Fullagar C. J., Mills M. J. (2010). Feeling good and doing great: the relationship between psychological capital and well-being. *Journal of Occupational Health Psychology*.

[29] Luthans F. (2002). Positive organizational behavior: Developing and managing psychological strengths. *Academy of Management Executive*.

